# Supplementary figures and images for: Biopsy-based normalizations of gill monogenean-infected European catfish (Silurus glanis L., 1758) stocks for laboratory-based experiments
Source: PeerJ. 2024 Nov 14;12:e18288. doi: 10.7717/peerj.18288 (PMC11569781; doi:10.7717/peerj.18288)

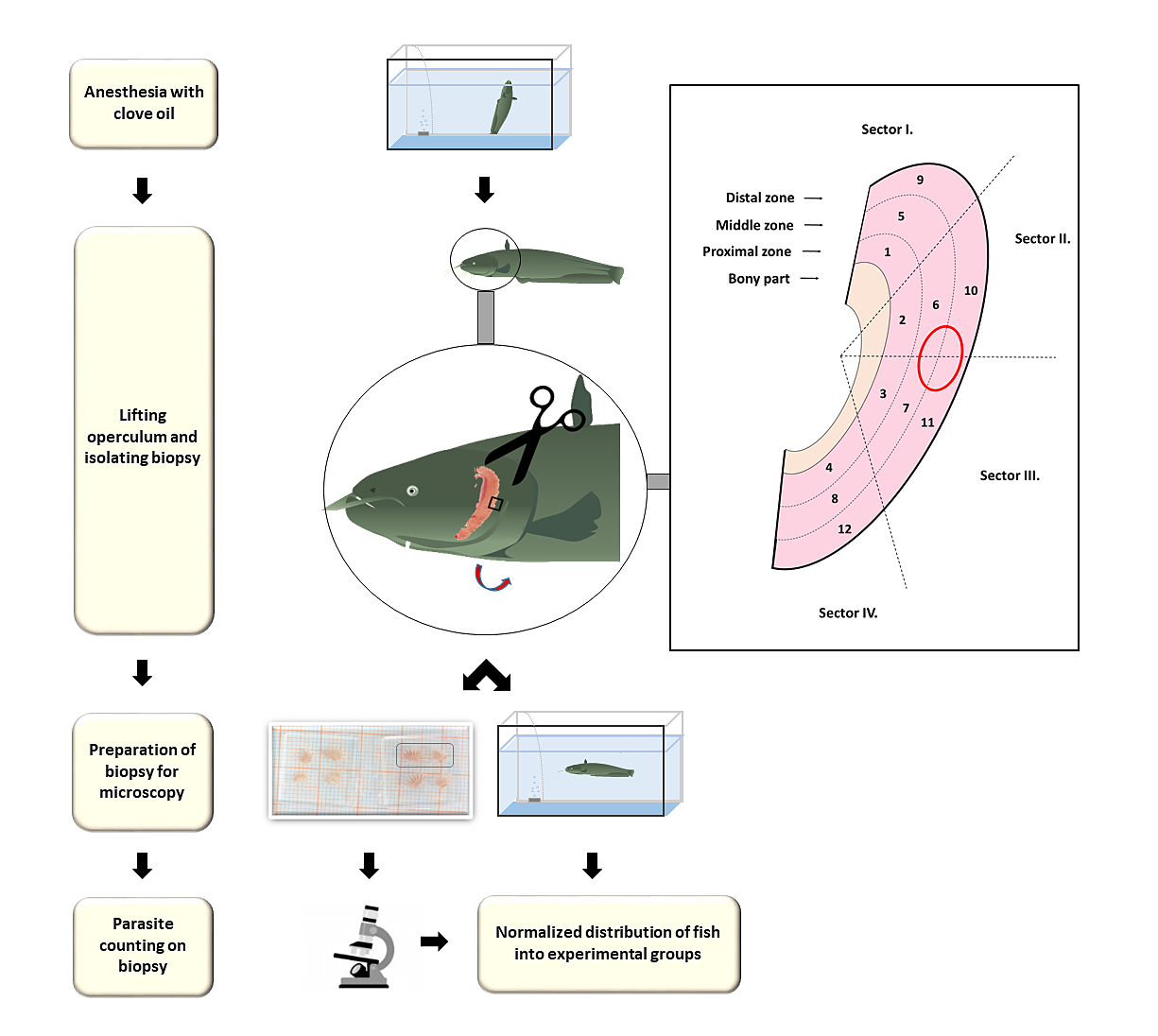

Supplement: Supplemental Information 3 — Anesthesia of catfish infected with T. vistulensis (Siwak, 1932) was performed in a separated tank using aquarium water mixed with clove oil. Inside the gill chamber an almond shaped, circa 3 × 3 mm biopsy incision was performed from the lamellae on the left first gill holobranch, encompassing 6-7-10-11 sectors involving both lamellae, resulting in two tissue pieces. The fish was then transferred into a different tank, while the parasite number was determined from the gill biopsy tissues. The parasite number obtained from the biopsy allowed for improved sorting of fish into batches. The components of the figure are original artwork. Gill partitioning graphics is presented according to Lo and Morand [29]. [file peerj-12-18288-s003.png]
